# Intrusion of Maxillary Posterior Teeth by Skeletal Anchorage: A Systematic Review and Case Report with Thin Alveolar Biotype

**DOI:** 10.3390/jcm11133787

**Published:** 2022-06-30

**Authors:** Avram Manea, Cristian Dinu, Mihaela Băciuţ, Smaranda Buduru, Oana Almășan

**Affiliations:** 1 Department of Maxillofacial Surgery and Implantology, Iuliu Hațieganu University of Medicine and Pharmacy, 37 Iuliu Hossu Street, 400029 Cluj-Napoca, Romania; avram.manea@umfcluj.ro (A.M.); mbaciut@umfcluj.ro (M.B.); 2 Prosthetic Dentistry and Dental Materials Department, Iuliu Hațieganu University of Medicine and Pharmacy, 32 Clinicilor Street, 400006 Cluj-Napoca, Romania; smarandabudurudana@gmail.com (S.B.); oana.almasan@umfcluj.ro (O.A.)

**Keywords:** maxillary posterior tooth intrusion, skeletal anchorage, orthodontics, thin alveolar bone

## Abstract

This study aimed to review the literature related to the intrusion of maxillary posterior teeth in subjects needing pre-prosthetic restoration or orthodontic treatment due to anterior open bite, and to report a thin alveolar biotype case needing a pre-prosthetic intrusion of maxillary teeth by introducing a novel, personalized method of intrusion measurement. An electronic search was conducted between February 2022 and March 2022 in the following databases: PubMed, Scopus, Embase, Web of Science, and Lilacs; the terms “tooth movement techniques”, “orthodontic anchorage procedures”, “tooth intrusion”, “intrusion”, “molar”, “premolar”, and “human” were surveyed. Eighteen articles were included in this review; the mean amount of intrusion ranged from between 2.1 ± 0.9 mm and 4.57 ± 0.98 mm (being mostly 2–3 mm). The intrusion force varied between 100 and 500 g; 10 articles reported miniscrews (MS), 7 reported zygomatic plates (ZP), and 1 publication reported both anchorage types. The average treatment time was 6.9 months for MS and 7.9 months for ZP. Levelling the occlusal plane by intrusion of the upper posterior teeth can be achieved by skeletal anchorage. The stability of the obtained results, shortening treatment time, and controlling treatment outcome are the main goals for a complex surgical and orthodontic treatment approach.

## 1. Introduction

Levelling the occlusal plane remains one of the major concerns in dentistry, especially in adult patients, due to the complex and multidisciplinary approach, as well as the skeletal component of the condition. Intrusion of the maxillary posterior teeth needs to be performed for open bite correction [1], or for prosthetic reasons, in order to level the occlusal plane due to overerupted molars attributable to post-extraction consequences. The true molar intrusion was considered rendered when the reference point to quantify the vertical movement of the molar in the dentoalveolar bone was the center of resistance of the tooth [2].

Occlusal interferences and functional disturbances may result in difficulties during prosthetic reconstruction [3]. Levelling the occlusal plane, including occlusal equilibration, is needed in cases with overerupted upper posterior teeth. This can be accomplished by root canal therapy with dental reshaping and prosthetic treatment [4], or by orthodontic intrusion using skeletal anchorage [5,6], surgical assisted impaction using corticotomy [7], or orthodontic surgery [8], ranging to much more extensive surgery, such as a LeFort I osteotomy with maxillary rotation [9]. A more frequent surgical technique in such cases is represented by the lateral maxillary segmental osteotomy, followed by the apical repositioning of the bone fragment [10]. This way, the intrusion effect is achieved instantly. However, the disadvantages associated with this technique (extensive surgery with the inherent postoperative discomfort, the need for a surgical splint) often convince the patient to decide in favor of a less invasive technique.

Temporary anchorage devices (TADs) represent an orthodontic treatment option, which is minimally invasive and aids in molar intrusion without needing the patient’s compliance [11]. Miniscrews, or miniplates, usually placed in the zygomatic buttress, can be used as TADs; molar intrusion obtained by skeletal anchorage is preferred compared to jaw surgery in severe open bite cases [12]. From a surgical point of view, miniscrew efficiency depends on bone density and soft tissue health [13]. The greatest amount of alveolar bone is located in the maxilla between the second premolar and the first molar [14]. Placement for the insertion of the miniscrews is influenced by the malocclusion and the quality and amount of appropriate bone, particularly in the interdental root space [15].

In adult patients, one of the most challenging malocclusions to correct with orthodontic treatment is anterior open bite [16], as this requires a complex multidisciplinary approach which draws on both surgical and orthodontic approaches. Treatment alternatives comprise molar intrusion, incisor extrusion, and maxillary impaction. A surgical approach, such as corticotomy, may aid in molar intrusion, limiting treatment time [17], although there are complications related to this strategy.

In performing an intrusive movement, the relationship between the maxillary posterior root apices to the inferior wall of the sinus should be considered, since the cortical bone layer of the maxillary sinus wall could represent a barrier to the intrusion [18]. Cone-beam computed tomography (CBCT) provides an accurate evaluation of the maxillary bone quality and quantity around the root apices of posterior teeth [19]. There is a current lack of studies evaluating true molar intrusion. A systematic review, due to its methodological rigor, represents evidence-based medicine when referring to unbiased knowledge syntheses [20].

To the best of our knowledge, a review related to the pre-prosthetic and orthodontic intrusion need of the maxillary posterior teeth has not yet been published. The aim of this study was to review the literature related to the intrusion of maxillary posterior teeth in subjects needing pre-prosthetic restoration or orthodontic treatment due to anterior open bite, and to report a thin alveolar biotype case needing pre-prosthetic intrusion of maxillary premolars and molars in order to develop a customized maxillary plane to propose a novel, personalized method of measuring intrusion. The clinical significance arises from the belief that the new palatal plane is simple to construct, assists in aligning the maxilla parallel to a specified reference line, and can also be performed in segmental CBCT images.

## 2. Materials and Methods

This review was performed following the recommendations of the “Preferred Reporting Items for Systematic Reviews and Meta-Analyses Protocols (PRISMA) Statement” [21].

### 2.1. Information Sources

A structured electronic search was conducted between February 2022 and March 2022 in the following databases: PubMed, Scopus, Embase, Web of Science, and Lilacs. Additionally, MeSH and Emtree terms were used, where applicable. Finally, a handsearching of relevant studies was performed.

### 2.2. Search Strategy 

The research strategy was constructed on the PICO framework (P—patient; I—intervention; C—Comparison; O—Outcome), as follows: P—patients with extrusion of upper posterior teeth; I—intrusion; C—no intervention; O—the amount of intrusion [22]. 

The terms “tooth movement techniques”, “orthodontic anchorage procedures”, “tooth intrusion”, “intrusion”, “molar”, “premolar”, and “human” were surveyed. The retrieved publications were imported into and organized in the Rayyan online software [23]. This software permitted a structured organization of the publications. Additionally, an automatized removal of the duplicates was possible, after carefully reading and deciding if the highlighted publication was a real duplicate. Two researchers independently accomplished the search and performed the selection, with the “blind on” mode turned on, for eliminating selection bias. Any disagreements were resolved by discussion and consultation between them and with a third author. For the assessment of each publication, Microsoft Excel spreadsheets (Microsoft Office 365, MS, Redmond, WA, USA) [24] were assembled, using Zotero 6.0.6 software (Corporation for Digital Scholarship, previously Center for History and New Media at George Mason University) [25].

The following inclusion criteria were pursued: human subjects requiring maxillary posterior tooth intrusion (molar or premolar), due to pre-prosthetic reasons to anterior open bite malocclusion; intrusion performed by skeletal anchorage (miniscrews or zygomatic plate); no previous orthodontic treatment; no orthognathic surgery; no tooth extractions; no active periodontal disease; no associated pathologies; publications with available full text in English language. The following exclusion criteria were considered: patients with systemic diseases; metabolic bone disorders; surgical assisted maxillary posterior teeth intrusion; photobiomodulation or other intrusion aiding techniques; intrusion followed by distalisation with the same anchorage device; mandibular molar intrusion; orthodontic treatments which involved tooth extractions, distalisation, mesialisation or rapid palatal expansion; orthognathic surgery; case reports; literature reviews, abstracts, and animal studies.

## 3. Results

### 3.1. Data Collection

**Figure 1:**
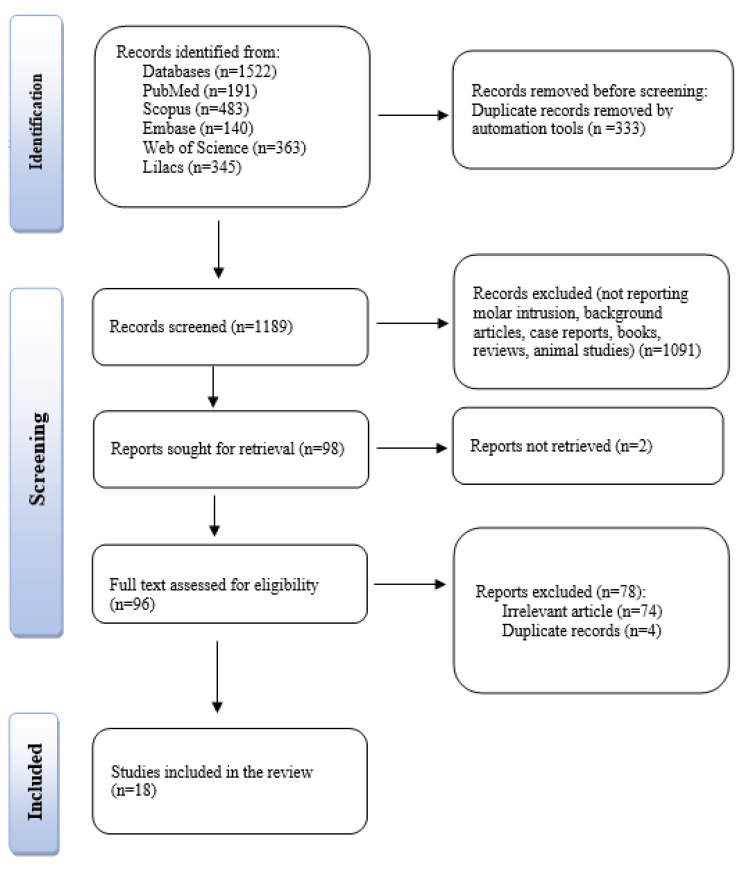
The PRISMA flowchart of the publication selection.

A total of 1522 records were identified, consisting of 191 from PubMed, 483 from Scopus, 140 from Embase, 363 from Web of Science, and 345 from Lilacs. After screening the duplicates, 333 records were excluded by automation tools. The titles, keywords, and abstracts of the remaining 1189 records were read, and 1091 records were excluded for not being related to the topic, or for not respecting the inclusion and exclusion criteria, as well as for being background articles, books, case reports, reviews, or animal studies. Ninety-eight records were sought for retrieval. Ninety-six articles were identified for eligibility, which met the inclusion criteria, and were checked for eligibility by full-text analysis. After careful reading and assessing the publications, a final number of 18 articles were selected and included in this review. The PRISMA diagram is shown in Figure 1.

### 3.2. Description of the Studies

Data were extracted using a standardized form, which included the following information: (1) authors’ names and publication year; (2) country; (3) aim of intrusion, (4) sample size, age range, and gender; (5) anchorage type; (6) intrusion measurement method; (7) intrusion range; (8) intrusion force; (9) treatment time; (10) outcomes; (11) side effects; and (12) conclusions.

Table 1 summarizes the basic characteristics of the publications evaluated in this research. Due to the heterogeneousness and the multiplicity of outcome measures among the included studies, meta-analysis was not achievable [26].

**Table 1:** Characteristics of the reviewed studies. Abbreviations are as defined as follows: IA—intrusion aim; IMM—intrusion measurement method; IR—Intrusion range; IF—intrusion force; TT—treatment time; OVE—overerupted; AOB—anterior open bite; MS—miniscrew, ZP—zygomatic plate, LC—lateral cephalogram, PAR—postero-anterior radiographs; PR—panoramic radiograph, CBCT—cone-beam computed tomography, NiTi—nickel-titanium; U6—upper first molar, PP—palatal plane; OB—overbite; FH—Frankfurt horizontal plane; T—trifurcation; PM—premolar; EARR—external apical root resorption; SN—sella to nasion plane, NA—not available.

Author, Publication Year	Country	IA	Sample Size, Age Range, Gender	Anchorage Type	IMM	IR	IF	TT	Outcomes	Side Effects	Conclusions
Akan B et al., 2020 [27]	Turkey	AOB	19 patients, (5 boys, 14 girls) 16.5 years	ZP, bilateral, acrylic appliance	LC	2.32 ± 2.13 mm	400 g, NiTi close coil springs	9.4 ± 0.7 months	U6 to PP occlusal plane OB anterior facial height	NA	“posterior dentoalveolar intrusion by zygomatic anchorage was an effective method for anterior open bite treatment”
Akl HE et al., 2020 [28]	Egypt	AOB	Intervention group: 10 subjects Control group: 10 subjects 18 to 25 years	4 MS: 2 infrazygomatic and 2 palatal	CBCT	Intervention group: 2.26 ± 1.87 mm Control group: 2.42 ± 2.06 mm	intervention group: 400 g NiTi closed coil springs control group: 200 g	6 months	U6 T or PM center to FH OB	Soft tissue overgrowth loose of two miniscrews	“no statistically significant difference in the amount of posterior teeth intrusion between 200 g and 400 g of applied intrusive force” “amount of intrusion increased gradually as the tooth was located more posteriorly, closer to the line of traction”
Al-Falahi B et al., 2018 [29]	Egypt	AOB	15 patients (13 females and 2 males), 14.5 to 22 years (mean age 18.1 ± 2.03 years)	MS, buccal	CBCT	2.79 ± 0.46 mm	300 g, elastomeric chain	5.1 ± 1.3 months	U6 to PP	EARR	“all evaluated teeth had statistically significant EARR; but, because of its small magnitude, it should be considered as clinically irrelevant”
Ari-Demirkaya A et al., 2005 [30]	Turkey	AOB	Study group: 16 (13 females, 3 males) 19.25 years (range 14–26 years) subjects control group: 16 subjects 19.43 years (range 14–25 years)	ZP	PR	NA	NA, closed Ni-Ti coil springs	NA	U6 tooth length	EARR	“apical root resorption of maxillary first molars after intrusion was done using zygomatic miniplates as skeletal anchorage was not clinically significantly different from apical root resorption associated with fixed orthodontic treatment without intrusion mechanics”
Ding WH et al., 2015 [31]	China	AOB	36 patients: 18 hyperdivergent 18 hypodivergent females (aged 20–42 years (28.93 ± 7.55	MS, buccal	CBCT	Hyperdivergent: 4.57 mm ± 0.98 Hypodivergent: 3.64 mm ± 1.25	100 g, elastomeric chains	Hyperdivergent: 3.13 months ± 0.90 Hypodivergent: 4.71 months ± 1.50	Difference of U6 distal buccal cusp-FH plane (DB-FH) + mesial buccal cusp-FH plane (MB-FH)/2	Miniscrew implants loose difference and change of bone during intrusion	“absolute molar intrusion could be achieved by miniscrew implant... more easily in hyperdivergent”
Heravi F et al., 2011 [32]	Iran	AOB	10 females (mean age 43.6 years, range 25 to 57 years)	MS, buccal, and palatal	Parallel periapical radiographs	2.1 ± 0.9 mm	100 g, occlusal arm with a force gauge hook	7.7 months (range: 4.3 to 11.5 months)	A reference axis of 2 landmarks in adjacent teeth a perpendicular line from this axis to each root apex	Dull pain on the day after surgery tongue irritation root resorption (mean 0.2 mm) intrusion relapse	“there was a significant correlation between treatment duration and mesiobuccal root resorption. No significant correlation was found between patient age and the amount of root resorption and intrusion”
Kim K et al., 2018 [33]	Korea	AOB	21 patients (3 men, 18 women); mean age 23.9 years (range 18.5– 36.4)	MS, buccal, and palatal	LC	2.2 ± 0.8 mm	NA	9.7 ± 3.2 months (range, 6.2–15.2 months)	U6 to PP	NA	“mandible exhibited counterclockwise rotation after maxillary molar intrusion; the center of mandibular autorotation was located behind and below condylion with individual variations” “the amount of molar intrusion demonstrated relationships with vertical and sagittal cephalometric parameters”
Li W et al., 2013 [34]	China Australia	OVE U6	12 patients (4 male; 8 female) 18 to 32 years, mean age: 24.3 ± 1.26 years	MS, buccal, and palatal	CBCT	3.3 ± 1.6 mm	150 g, elastic chain	6 ± 1.59 months; range: 4 to 9 months	Crown’s central fossa to reference plane	Root resorption	“volume measurements using CBCT could effectively evaluate the root resorption caused by mini-screw intrusion”
Marzouk ES et al., 2015 [35]	Egypt	AOB	13 patients (9 females; 4 males) mean age 18 years, 8 months ± 2 years, 2 months	ZP	LC	3.1 ± 0.74 mm (range: 2–4 mm)	450 g, NiTi closed coil spring	9 ± 2.5 months	U6 to PP	NA	“intrusion of the posterior teeth with skeletal anchorage induced counterclockwise rotation of the mandible”
de Oliveira TFM et al., 2015 [36]	Brazil	AOB	9 patients (6 females, 3 males; mean age 18.7 ± 5.1 years)	ZP	LC oblique radiographs at 45°	2.03 ± 0.87 mm	450–500 g, elastomeric chains	6 months	Anteroposterior position of the molar cusp and root apex The vertical position of the molar cusp and root apex	Possible root resorption	“skeletal anchorage provided intrusion of molars without changing the palatal plane angle”
Paccini JV et al., 2016 [37]	Brazil	OVE U6	19 patients (4 males, 15 females) Group 1: mean age 34.25 years ± 8.22 (range: 22.66–46.99) Group 2: mean age 39.47 years ± 8.12 (range: 21.07–47.44)	MS group 1: 2 MS: 1 buccal,1 palatal group 1: 3 MS: 2 buccal,1 palatal	LC	Group 1: 1.79 ± 1.28 mm Group 2: 2.12 ± 1.25 mm	150 g, elastomeric chain	Group 1: 0.81 years ± 0.5 (range 0.41–1.64 years) Group 2: 1.17 years ± 0.48 (range 0.75–2.14 years)	U6 to PP U6 to SN OB	NA	“protocols of maxillary molar intrusion with two or three mini-implants presented the same efficiency of skeletal anchorage”
Pinzan-Vercelino CRM et al., 2015 [38]	Brazil	PP	9 patients (7 females, 2 males) mean age 37.17 years (range: 28.5–46.41	MS, buccal, and palatal	LC	Mean 2.4 mm (range: 1.2–4.5 mm)	NA	9.03 ± 4.04 months (range: 3.16–16.23 months)	U6 to PP	NA	“orthodontic intrusion using direct anchorage of mini-implants was an effective method for the intrusion of maxillary molars”
Scheffler NR et al., 2014 [39]	USA	AOB	30 patients (11 male and 19 female)	16 patients MS, buccal 14 patients ZP	LC	2.3 mm	NA, NiTi coil springs occlusal splint		Anterior face height mandibular plane angle OB	relapse no failures of miniplate anchorage 1 loose MS 1 MS fell out	“intrusion of the maxillary posterior teeth can give satisfactory correction of moderately severe anterior open bites, but 0.5 to 1.5 mm of reeruption of these teeth is likely to occur”
Seres L, Kocsis A, 2009 [40]	Hungary	AOB	7 patients (4 women and 3 men), mean age 21 years (range, 15–29 years)	ZP	LC, PR, periapical radiographs	NA	100 to 120 g, NiTi closed coil springs	6 months	Mandibular plane closed Point B rotated anteriorly and upward	Mild discomfort after surgery No signs or symptoms of a temporomandibular dysfunction were observed, No miniplate movement was detected no significant root resorption	” skeletal anterior open bites due to posterior maxillary dentoalveolar hyperplasia can be closed without orthognathic surgery”
Sherwood K.H. et al., 2002 [41]	USA	AOB	4 patients (2 men and 2 women)	ZP	LC, PR	Mean: 1.99 mm Range: 1.45–3.32	Coated elastic thread	5.5 months	2 measurement lines on PR anterior facial height mandibular plane occlusal plane	No discernable movement of any miniplate	“true intrusion of molars can be accomplished in adults” “Anterior open bites can be closed by intruding posterior teeth, resulting in reduced anterior vertical face height, decreased mandibular plane angle, and counterclockwise rotation of the mandible”
Turkahraman H., Sarioglu M, 2016 [42]	Turkey	AOB	40 patients: 20 treatment group (14 female, 6 male) mean age: 16.68 ± 2.80 years 20 control group (11 female, 9 male) mean age: 16.63 ± 2.83 years	ZP	LC	Treatment group: 3.59 ± 1.34 mm control group: 0.51 ± 0.44 mm	200 g Ni-Ti coil springs	Treatment group: 1.00 ± 0.31 years control group: 0.95 ± 0.14 years	U6 to PP	Mesial movement of the molars by 1.52 mm was found in the treatment group	“mild to moderate skeletal anterior open bites could easily be treated with TADs without orthognathic surgery. With the rigid anchorage of mini plates, true molar intrusion was achieved”
Xun CL et al., 2013 [43]	China	OVE U6	30 patients 35.5 ± 9.0 years (range 19 to 50)	MS	LC. PR	3.4 mm (range 1.5 to 6.5 mm)	100–150 g, elastic chain	6.2 ± 2.1 months	U6 to PP	Crown of the molars mesially tilted by averages of 3.1 degrees root resorption 0.2–0.4 mm on average	“intrusion treatment of over erupted molars with miniscrew anchorages could be used as an efficient and reliable method to recover lost restoration space for prosthesis”
Yao CC, et al., 2005 [3]	Taiwan	OVE U6	22 patients mean age 27.6 years (range: 15 to 42 years)	MS	Dental casts	mean: 3.1 ± 1.7 mm (range 0.34 to 8.67 mm)	150–200 g, elastic chain	7.6 months (range 5–12 months)	Three-dimensional (3D) digitizer, superimposing two sets of data to assess the relocation of cusp tips	Buccal–lingual tipping of the intruded U6 Clinical crown shortening of the intruded teeth	“a combination of mini-implants and fixed appliances is a a predictable and effective procedure to achieve maxillary molar intrusion”

### 3.3. Study Characteristics

Eighteen publications were evaluated in this review. In terms of publishing country, three of them were from Brazil, Egypt, and Turkey, respectively; two were from China and the USA, respectively, while there was one from China and Australia, Hungary, Iran, Korea, and Taiwan, respectively. Thirteen studies aimed at intruding upper first molars due to anterior open bite, whilst in the other studies the objective was the correction of the overeruption of the first molar, and just one study clearly stated the pre-prosthetic reason for intrusion. The vast majority of research that targeted correcting intrusion for open bite included participants aged between 18 and 30 years, whereas in the studies which aimed at intruding molars for overeruption, ages ranged between 20 and 46 years. The mean age, among the studies that reported it (15 studies) was 26.475 years. Regardless of the intrusion goal, there was a gender difference, with females being more prevalent. Ten studies used miniscrews (MS) as the anchorage type, seven used a zygomatic plate (ZP), and one publication used a combination of MS and ZP. In some of the publications, the reported method of intrusion technique was nickel-titanium (NiTi) coil springs [27,28,30,35,39,40]. Other authors reported the use of elastomeric chain [3,31,34,36,37,43].

The mean amount of intrusion was similar across studies, with a range of between 2.1 ± 0.9 mm and 4.57 ± 0.98 mm, being mostly situated between 2–3 mm. The intrusion force varied between 100 and 500 g and, although most of the studies (n = 8) reported a force between 100 to 200 g, one study reported 300 g [29], and four studies reported an intrusion force between 400 to 500 g [27,28,35,36]. In five of the studies, we could not identify the amount of force used.

The intrusion amount was measured in lateral cephalograms (LC) in eight studies, lateral cephalograms (LC) and panoramic radiographs (PR) in two studies, lateral cephalograms (LC), panoramic radiographs (PR) and periapical radiographs in one study, CBCT scans in four studies, only panoramic radiographs (PR) in one study, parallel periapical radiographs in one study, and dental cast models also in one study. Eight of the eighteen papers measured the distance between U6 to PP, while the others used mixed methods or custom measurement techniques. 

The treatment time was reported in 16 studies, ranging from 3 to 12 months, with a mean value of 7.56 months.

Thirteen studies reported side effects, while the other five mentioned no issues during or associated with the intrusion. One paper reported soft tissue overgrowth, seven articles described external apical root resorption (EARR) of various degrees, and three studies reported mini-screw loosening. Relapse appeared to be an issue in one study, different degrees of post-surgical discomfort and tongue irritation was mentioned in two studies while three studies reported coronal tilting and other unwanted movements, accompanying the intrusion process. 

All the articles state the fact that TADs are an efficient treatment option for obtaining a correction of either anterior open bite or levelling of the occlusal plane, with minor side effects if any, and, more importantly, reducing the need for much more invasive and complex interventions, such as orthognathic surgery.

### 3.4. Risk of Bias in Studies

The risk of bias was assessed according to the Newcastle–Ottawa Quality Assessment Scale for case-control studies to evaluate the methodological quality of the selected publications [44] (Table 2). According to this scale, each numbered item in the “selection” and “exposure” categories could yield a maximum of one star, whereas “comparability” could receive a maximum of two stars.

### 3.5. Case Report 

**Figure 2:**
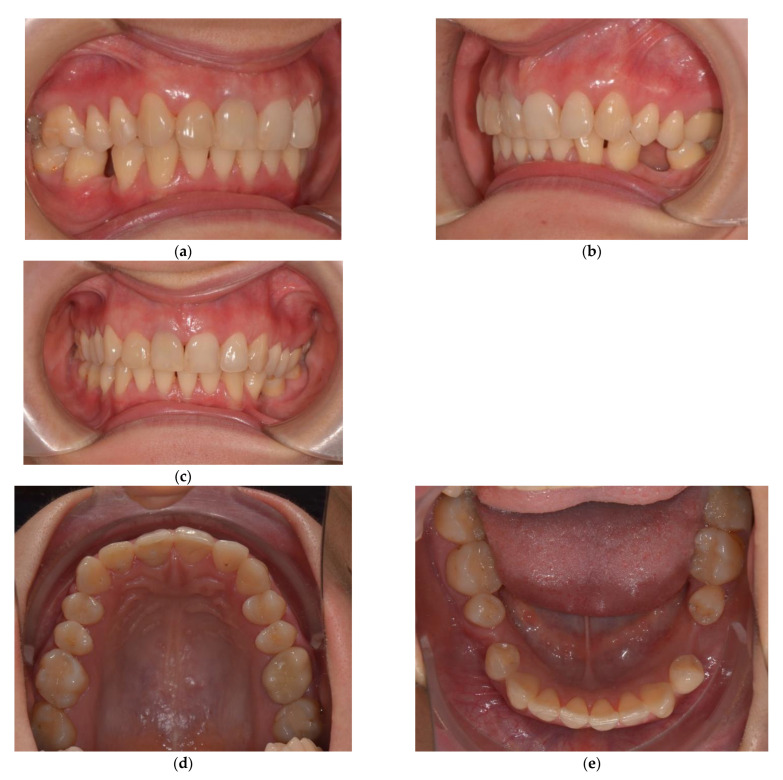

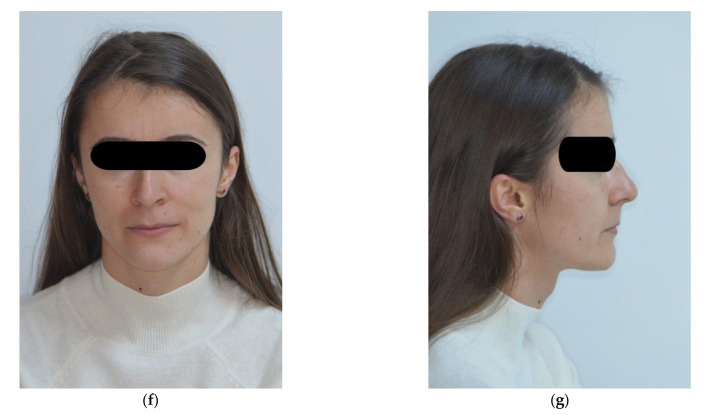
Initial situation (**a**) Right lateral occlusal view; (**b**) Left lateral occlusal view; Initial situation (**c**) Frontal occlusal view; Initial situation (**d**) Upper arch; (**e**) Lower arch; Initial situation, extraoral photos (**f**) Frontal view; (**g**) Lateral view.

A 28-year-old woman, seeking replacement of missing lower first molars, with second mandibular premolars shifted distally and rotated to the edentulous space, came to our practice. Prosthetic treatment of the edentulous spaces was limited by overeruption of upper first molars and premolars, as well as by the rotated teeth. The occlusal plane was irregular, with extrusion of the upper first molars and premolars into the edentulous spaces and a lower midline shift of 2 mm towards the right side (Figure 2). She had a hypodivergent skeletal pattern, a class II skeletal pattern with a small anterior facial height, skeletal deep bite tendency, and increased overbite and overjet (Table 3). 

**Figure 3:**
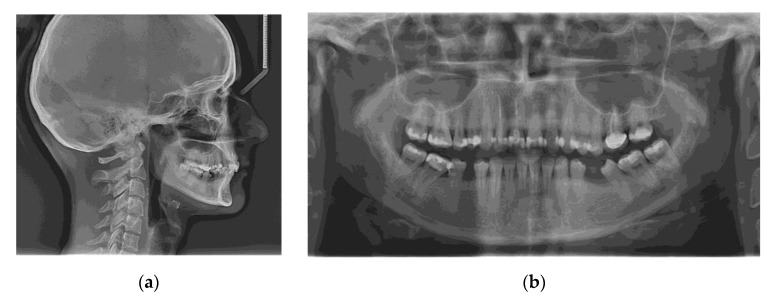
Radiographic examination before treatment: (**a**) Initial lateral cephalogram; (**b**) Initial panoramic radiograph.

The initial radiographs are shown in Figure 3. No signs of periodontal disease or other associated pathologies were encountered on panoramic radiography.

The main treatment objectives included obtaining a functional occlusion, intruding the maxillary first molars and premolars, levelling the occlusal plane, creating space for prosthetic replacement of the lower molars, achieving functional arch relationships, and enhancing masticatory efficiency. The treatment plan involved orthodontic intrusion of the overerupted upper teeth, followed by fixed appliance therapy. On the right hemiarch, one miniscrew on the buccal side and two on the palatal side were placed, whereas on the left hemiarch, a zygomatic plate was placed on the buccal area, along with two miniscrews on the palatal area. 

In the first quadrant, four mini screw implants, temporary anchorage devices (TAD) were inserted, as follows:

- 12 mm (Ø 1.6 mm) Jeil Dual Top JA Screw–Palatal, between tooth 1.3 and 1.4
- 12 mm (Ø 1.6 mm) Jeil Dual Top JA Screw–Buccal, between tooth 1.4 and 1.5
- 12 mm (Ø 1.6 mm) Jeil Dual Top JA Screw–Buccal, between tooth 1.6 and 1.7
- 12 mm (Ø 1.6 mm) Jeil Dual Top JA Screw–Palatal, distal of tooth 1.7

**Figure 4:**
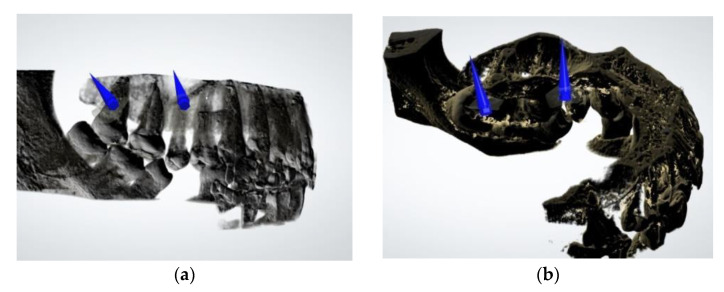
Preoperative surgical planning: (**a**) Buccal miniscrews; (**b**) Palatal miniscrews.

All the TADs were inserted under local anesthesia (Ubistesin Forte, articaine hydrochloride 4% with adrenaline (epinephrine) 1:200,000, 2 × 1.7 mL). The preoperative planning aimed for bicortical anchorage (confirmed by postoperative CBCT scan, Figure 4).

The TADs were inserted using the NSK Surgical Pro Fiziodispenser and NSK Ti-Max Contra Angle Handpiece (20:1 Reduction), using a rotation speed of 30 rpm and a 30 N/cm insertion torque. No prior preparation of the insertion site was required. 

**Figure 5:**
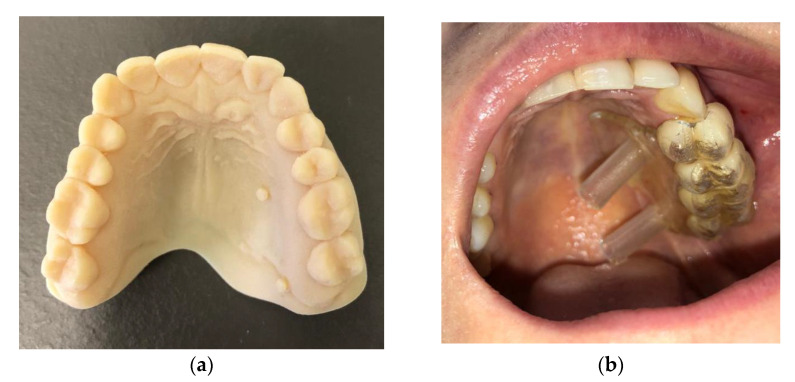
Surgical guide for the two screws in the second quadrant: (**a**) 3D printed cast; (**b**) Surgical guide applied in the oral cavity.

In the second quadrant, two mini screw implants were inserted in the palatal region (12 mm (Ø 1.6 mm) Jeil Dual Top JA Screw), the first one between tooth 2.3 and 2.4 and the second distal to tooth 2.7, using the same protocol as for the first quadrant. The only difference consisted of the use of a surgical guide for the two screws in the second quadrant (Figure 5). A printed 3D model was obtained using the patient’s preoperative scan with a Formlabs Form 3 printer.

In addition, due to reduced bone volume compared to the first quadrant and reduced interproximal space, an orthodontic anchor plate was chosen for the buccal area, which was customized on the 3D model. 

**Figure 6:**
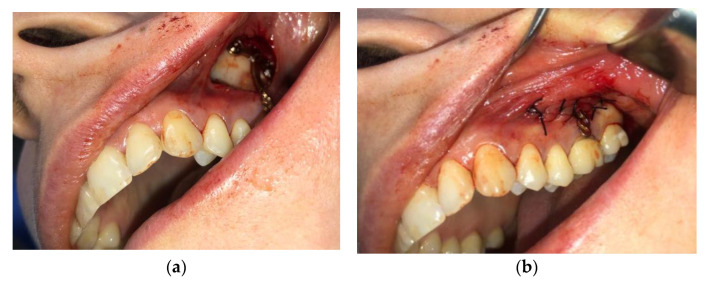
Zygomatic anchorage on the left maxillary buccal area: (**a**) with the exposure of the zygomaticomaxillary buttress; (**b**) Nonresorbable 4/0 Supramid simple interrupted suture.

For this region, the same type of local nerve block was used. An incision was placed at the mucogingival junction starting from tooth 2.7 to tooth 2.3, followed by the elevation of the mucoperiosteal flap, with the exposure of the zygomaticomaxillary buttress (Figure 6). The plate was sterilized after personalization and before surgery. This preoperative step dramatically reduces surgery time and provides perfectly predictable results. The plate was anchored to the zygomaticomaxillary buttress using three 2.0 self-tapping screws. Nonresorbable 4/0 Supramid simple interrupted sutures were used, and these were then removed seven days after surgery.

To calculate the amount of performed molar intrusion, the difference of the linear distance from the mesiobuccal cusp of the maxillary first molar to a custom palatal plane (CPP) was measured on CBCT images before and after intrusion mechanics. The CPP was defined by the following three points: ANS, and the lowest points of the pterygoid hamulus on the left and right sides. The measurements were performed by one maxillofacial surgeon and one orthodontist, twice, and mean values were considered (Table 4).

**Figure 7:**
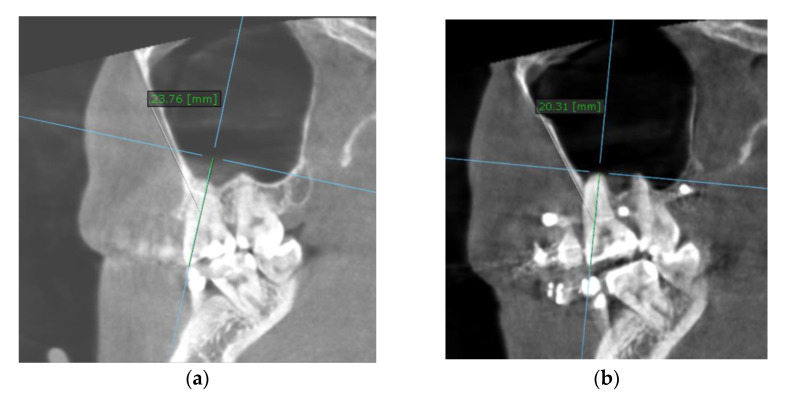
Intrusion amount, at the level of the mesiobuccal cusp of the right upper first molar (the distance between the cusp tip and CPP): (**a**) before intrusion: 23.76 mm; (**b**) after intrusion: 20.31 mm.

The measurement method is shown in Figure 7.

The left zygomatic plate and miniscrews were placed initially, and intrusion mechanics began with the aid of an elastic chain on this side after two weeks of soft tissue healing. Subsequently, at approximately 1.5 months after the left maxillary arch, the right hemiarch was implanted and miniscrews were inserted. No associated symptoms were described by the patient, and no signs of tissue irritation were found. No loose miniscrews or other accidents occurred. 

Elastomeric chains were changed every four weeks. Approximately 3.59 mm of intrusion was achieved on the right buccal side and 2.21 mm on the right palatal side in six months, 2.26 mm on the left buccal side, and 1.86 mm on the left palatal side in nine months. After intrusion, ligature stainless steel wires were used to keep the intruded teeth in place. Subsequently, upper and lower teeth were included in a full arch appliance, with a 0.022 MBT prescription. 

**Figure 8:**
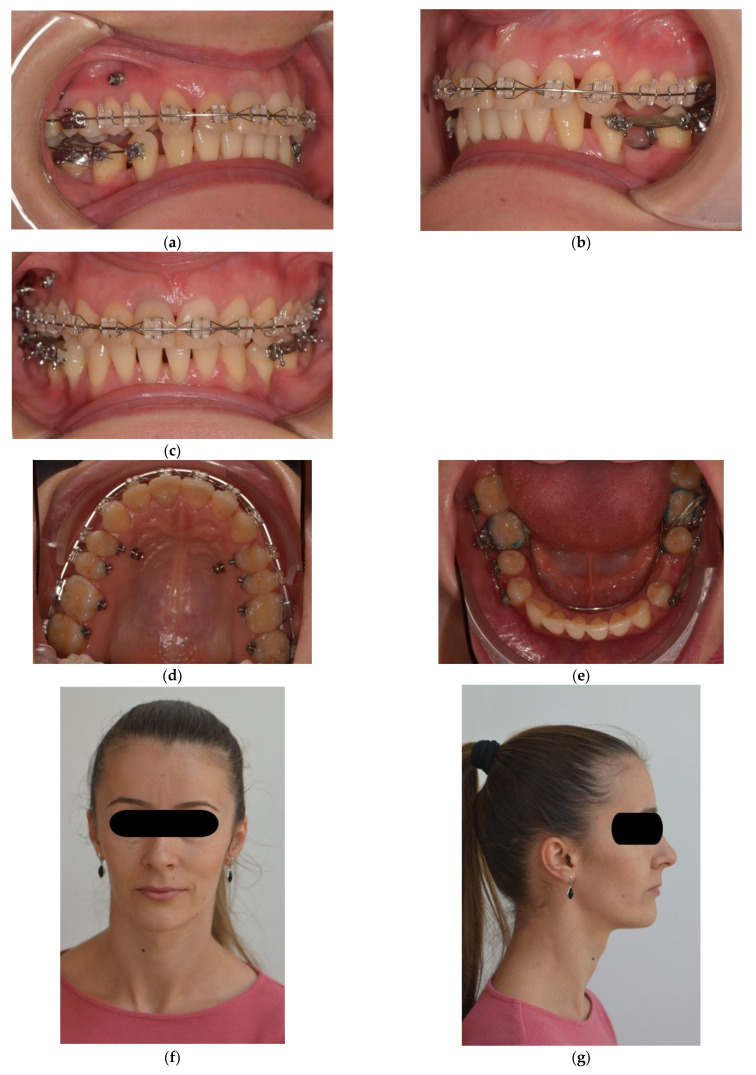
Post-intrusion intraoral photographs (**a**,**b**) Left lateral occlusal view; (**c**) Frontal occlusal view; (**d**) Upper arch; (**e**) Lower arch; Post-intrusion intraoral extraoral photos (**f**) Frontal view; (**g**) Lateral view.

The main aim of the treatment objectives, namely the intrusion of the upper posterior teeth, has been achieved. The orthodontic treatment is ongoing in order to solve additional objectives, such as midline correction and space distribution for prosthetic treatment (Figure 8).

## 4. Discussion

We initially searched for intrusion aims, such as pre-prosthetic molar or premolar intrusion, as well as an orthodontic intrusion for open bite correction. We only found a few papers linked to the pre-prosthetic intrusion goal, most of which were case reports, hence, they were not included in this review. Nonetheless, a few articles on the subject were found. There are a few reviews related to intrusion for open bite correction, but none of them focused on both orthodontic and pre-prosthetic aspects. Furthermore, non-orthodontic cases of overerupted molars are rare, and the number of included subjects is small.

Sherwood et al., reported just four cases, treated for anterior open bite [41]. The largest number of cases, 36, was reported by Ding et al., also for anterior open bite treatment [31]. The publications aiming at intruding overerupted upper molars for prosthetic reasons included 9 subjects [38], 12 subjects [34], 19 subjects [37], 22 subjects [3], and 30 subjects, respectively [43]. 

Adults with overerupted molars because of antagonist loss are still a common clinical finding. Occlusal plane rehabilitation should use a multidisciplinary approach. Molar intrusion is required to provide adequate space for prosthetic rehabilitation. If possible, the implant location should be chosen based on the availability of sufficient cortical bone [45]. Due to the maxillary sinus and the thin alveolar biotype, we encountered risks in the intrusion mechanism and miniscrew placement, prolonging the intrusion time.

The recommended loading force of the anchorage devices has been suggested to vary between 50–500 g, reported as 50 g [46,47], 100–200 g [48,49], or 300–500 g of force [50]. If intrusion of more than one single tooth is needed at the same time, the force should be higher, around 400 g [51]. The recommended amount of intrusion of the overerupted maxillary molars is approximately 0.5–1.0 mm per month, without the occurrence of unwanted secondary effects, such as root resorption, periodontal effects, or vitality loss [11]. In the reviewed publications, intrusion force varied between 100 and 500 g. The use of skeletal anchorage can aid in an increased amount of molar intrusion, allowing for accelerated orthodontic forces [52]. Nonetheless, we encourage close monitoring to minimize the risk of undesired side effects. In most studies, the amount of intrusion was obtained by measuring the distance between a reference point on the first molar and the palatal plane on lateral cephalograms. Most of the available studies using 3D imaging measure distance from various tooth landmarks to the palatal plane, defined as passing through anterior nasal spine (ANS), posterior nasal spine (PNS), and perpendicular to the mid-sagittal plane [29]. Baek et al., using 2D imaging, defined the plane as crossing through the ANS and PNS [53]. Although this study met most of our inclusion criteria, it was excluded due to the fact that the study protocol included extractions. Although 2D measurements are easier to perform and more reproducible, very few parameters can be evaluated, leaving 3D imaging as the most precise and relevant alternative. Due to the uncertainty of the definition of the mid-sagittal plane, selecting this landmark might be a source of bias since various factors can influence measurements from pre-treatment and post-treatment CBCT scans. This is the reason why, for the present case report, we decided to define a custom plane, that can always be reproduced with maximum accuracy. This plane is defined by ANS and the lowest points of the pterygoid hamulus on the left and right sides. 

Out of the 18 reviewed articles, 10 reported MS, 7 reported ZP, and 1 used both anchorage types. The average treatment time was 7.1 months for those using MS, and 7.9 months for the ones that had ZP, which follows the results presented in our case. This might lead to the conclusion that the intrusion using MS could be quicker. One must keep in mind, though, that ZPs are frequently used for the more severe cases, where the requirement for intrusion is increased, leading to the fact that efficiency cannot be evaluated solely based on the type of TAD. Additionally, it might be assumed that ZPs are used when broader movements are needed. However, within the findings of the present review, the average intrusion was similar between the two groups (2.6 mm for the ZPs and 2.7 mm for the MSs). The mean amount of intrusion reported in the selected articles was quite similar, being mostly between 2–3 mm. 

Everdi N et al., when intruding upper first molars with the aid of zygomatic plates and NiTi coil springs, reported an average intrusion of 2.6 mm, but the authors included patients with extractions. However, they found buccal tipping of the maxillary molars, as well as inflammation at the TAD site [54]. Various strategies for assessing molar intrusion have been described, depending on the methodology of assessment. It has been postulated by Burstone that true molar intrusion can only be verified when the molar’s center of resistance is utilized as a point of comparison to measure the molar’s vertical displacement into the alveolar bone [55]. In that regard, using other reference points, such as cusp tips or root apex, would make it difficult to distinguish intrusion from tipping [55]. It has been shown that the treatment of anterior open bite by molar intrusion, accomplished by reducing the distance between the mesial buccal cusp of the first molar and the palatal plane, can be achieved [56].

The intrusion time ranged from 3 [31] to 12 months [42], with the majority of publications (n = 8) reporting an average of 5 to 6 months, 2 studies of 7 months, and 5 publications of 9 months. However, due to the heterogeneity of the included studies, a relationship between anchorage type and time could not be revealed. The recommended intrusion rate for a single molar is 0.75 mm per month, whilst for the intrusion of grouped teeth (first molar and second premolar) it is 0.5 mm per month [54]. 

Molar intrusion has grown more successful and efficient because of skeletal anchoring, yet it is still considered a challenging orthodontic technique [6]. It is an effective method of intruding molars to address an open bite [52]. 

The relationship between arch bracketing and the intrusion mechanism has been intensely studied. It has been shown that when the arch is not braced, true posterior segment intrusion ensues [41]. However, the inferior wall of the maxillary sinus must be considered when intruding upper molars. The movement of posterior teeth across the maxillary sinus has been linked to moderate apical root resorption and increased tipping [57,58]. In our case, no movement of the zygomatic buttress miniplate or the buccal or palatal microscrews occurred neither during their use nor before clinical removal. The CBCT showed no discernible signs of root resorption. 

There are also some important risks and complications of TAD placement, as follows: root trauma, anchorage failure, sinus perforation, nerve injury, soft tissue irritation, relapse [11], contact of the TAD with the adjacent roots, miniscrew loosening or fracture, damage to anatomic tissues, soft tissue overgrowth [15]. Among the possible side effects, most studies reported external apical root resorption. Additionally, loose miniscrews, soft tissue irritations, relapse, mesial movement of molars, and tipping have been described. Although MSs were used more commonly, the complications were fewer in the ZP cases. Only one article showed certain EARR. On the other hand, several cases presented EARR in the MS group, along with soft tissue overgrowth and irritation and, most important of all, frequent MS loosening. Another advantage of the ZP TAD system is represented by the traction forces that it can withstand (an average of 327 g, compared to the average of 187 g for the MS group). 

True molar intrusion as described by de Oliveira et al., with no modifications of the anteroposterior orientation of molars, mesial tipping of posterior teeth, or no changes in the palatal plane angle [36], has been encountered as well in the reported case. The more common usage of MS shows the clinicians’ bias towards this type of TAD, due to their ease of application, low risks, and elevated success rate. Additionally, ZP were used more commonly when a translation of molars was needed to correct the malocclusion [39]. 

Since the approach of posterior teeth intrusion may offer predictable results without relying largely on patient compliance, orthodontic correction of the occlusal plane using a skeletal anchorage should be regarded as state-of-the-art. Nevertheless, due to a high degree of relapse, the intrusion must be maintained by retention procedures. Gonzales et al., have shown that, due to a high degree of recurrence, the stability of open bite treatment with molar intrusion employing skeletal anchoring in adult patients might be regarded as relatively unstable [59].

A dual assessment of the risk of bias was conducted by two authors (O.A. and A.M) to identify potential sources of bias, which is critical for future research quality. Although there was a moderate risk of bias in the selected studies, mainly due to the lack of controls, the flaws were not severe enough to invalidate the findings. The absence of untreated control groups, a short follow-up time, a small sample size, and the lack of intrusion force measurement in some articles were all found to be shortcomings in the reviewed publications. There is still little valid scientific research available to assess actual molar intrusion [2]. 

Among the limitations of this study might be the reduced number of cases included in the selected articles, the lack of controls, and the variability of used skeletal anchorage, intrusion force, and intrusion mechanism. Additionally, some authors did not report the intrusion amount, intrusion force, intrusion mechanism, or side effects. A major concern is the lack of an untreated control group, although some authors compared the intervention group to another treated group, but with different force amounts. Due to the heterogeneity of the publications, a meta-analysis could not be performed. 

A strong point of the present study, besides the thorough literature analysis regarding TADs, is the definition of a custom plane that can be used for exact measurements, to evaluate tooth intrusion. The classical PP can be affected by several patient- and device-specific factors, rendering the pre- and post-operative measurements ineffective. Measuring the distance from certain tooth landmarks to the CPP will always yield useful and relevant findings, since this plane is defined by anatomical landmarks that can not suffer changes throughout tooth ingression. 

Our future recommendations are as follows: a fundamental goal is to define a customized reference plane to ensure optimal reproducibility of pre-and post-surgical measurements. The CBCT scans must be performed under the same conditions, on the same equipment, and, if feasible, by the same operator to improve data consistency and reliability. Optimal anchorage and force management must be aimed, thus, reducing tooth movement (intrusion) time and limiting side effects.

## 5. Conclusions

According to the findings of this study, there is evidence that levelling the occlusal plane by the intrusion of the upper posterior teeth can be achieved by skeletal anchorage.

In the presented case it was possible to obtain a well-controlled intrusion of the maxillary molars and premolars without unwanted side effects. Stability of the obtained results, shortened treatment time, and controlling treatment outcome are the main goals for a complex treatment approach, which draws on both surgical and orthodontic practices. 

The manuscript’s strengths rely on a thorough analysis of the existing literature and the definition of a CPP (a custom palatal plane defined by ANS and the lowest points of the pterygoid hamulus on the left and right side), which aims to reduce the inaccuracy of true tooth intrusion evaluation.

To achieve the highest possible long-term outcomes, dental practitioners should be up to date on the latest technologies to be used as alternatives for specialized treatment planning and patient monitoring.

## Figures and Tables

**Table 2:** The Newcastle–Ottawa Quality Assessment Scale for case-control studies. *-fulfilled criteria.

Author, Year of Publication	Akan B et al., 2020 [27]	Akl HE et al., 2020 [28]	Al-Falahi B et al., 2018 [29]	Ari-Demirkaya A et al., 2005 [30]	Ding WH et al., 2015 [31]	Heravi F et al., 2011 [32]	Kim K et al., 2018 [33]	Li W et al., 2013 [34]	Marzouk ES et al., 2015 [35]
1. Is the case definition adequate?		*	*	*	*	*	*	*	*
2. Representativeness of the cases	*	*	*	*	*		*	*	*
3. Selection of controls		*		*	*				
4. Definition of controls		*		*					
1. Comparability of cases and controls on the basis of the design or analysis		*		*	*				
1. Ascertainment of exposure	*	*	*	*	*	*	*	*	*
2. Same method of ascertainment for cases and controls		*		*					
3. Non-response rate									
**Author, Year of Publication**	**de Oliveira TFM et al., 2015 [36]**	**Paccini JV et al., 2016 [37]**	**Pinzan-Vercelino CRM et al., 2015 [38]**	**Scheffler NR et al., 2014 [39]**	**Seres L, Kocsis A, 2009 [40]**	**Sherwood K.H. et al., 2002 [41]**	**Turkahraman H., Sarioglu M, 2016 [42]**	**Xun CL et al., 2013 [43]**	**Yao CC, et al., 2005 [3]**
Selection									
1. Is the case definition adequate?	*	*	*	*	*	*	*	*	*
2. Representativeness of the cases	*	*	*	*	*	*	*	*	*
3. Selection of controls							*		
4. Definition of controls							*		
Comparability									
1. Comparability of cases and controls on the basis of the design or analysis							*		
Exposure									
1. Ascertainment of exposure	*	*	*	*	*	*	*	*	*
2. Same method of ascertainment for cases and controls							*		
3. Non-response rate									

**Table 3:** Lateral cephalometric measurements.

Parameter	Value	Mean ± SD
SNA angle	84.94°	82 ± 2°
ANB angle	4.23°	2 ± 2°
SNB angle	80.71°	80 ± 2°
FMA angle	21.53°	25 ± 2°
Occlusal plane to Gonion–menton	13.83°	19.09 ± 4.7°
Occlusal plane to Sella–nasion	16.33°	14 ± 4°
Lower facial height	65.64 mm	66.7 ± 4.1 mm
Anterior facial height	114.80 mm	128.68 ± 6 mm
Upper molar to pterygoid vertical plane	21.39 mm	21.10 ± 3 mm
Interincisal angle	145.21°	128.0 ± 5°
Overbite	3.73 mm	2 ± 2 mm
Overjet	3.3 mm	2 ± 2 mm
Gonion–Gnation to Sella–nasion	28.91°	32 ± 4°
U1 to Nasion–point A line	10.27°	22.0 ± 5°
U1 to Sella–nasion	95.21°	105.28 ± 6°

SNA-sagittal position of the maxilla; SNB-sagittal position of the mandible; FMA-facial pattern; U1-upper incisor; S = sella point; N = nasion point; SD-standard deviation.

**Table 4:** The CBCT measurements before and after intrusion at the level of upper first molar and upper first premolar.

CBCT Parameter	T0–Before Intrusion (mm)	T1–After Intrusion (mm)	Intrusion Amount (T1-T0; mm)
Mesiobuccal cusp of the left upper first molar	22.26	20.73	1.53
Palatal root apex of the left upper first molar	4.20	1.62	2.58
Upper left first molar furcation	11.35	8.74	2.61
Buccal cusp of the left upper first premolar	24.8	22.16	2.64
Palatal root apex of the left upper first premolar	5.67	4.53	1.14
Mesiobuccal cusp of the right upper first molar	23.76	20.31	3.45
Palatal root apex of the right upper first molar	4.18	2.37	1.81
Upper right first molar furcation	12.79	9.74	3.05
Buccal cusp of the right upper first premolar	24.72	20.45	4.27
Palatal root apex of the right upper first premolar	6.04	3.42	2.62

## Data Availability

The data presented in this study are available from the corresponding author upon reasonable request.
